# Muscle Atrophy and mRNA-miRNA Network Analysis of Vascular Endothelial Growth Factor (VEGF) in a Mouse Model of Denervation-Induced Disuse

**DOI:** 10.7759/cureus.68974

**Published:** 2024-09-09

**Authors:** Gaku Oguri, Ryo Ikegami, Haruka Ugawa, Manami Katoh, Syotaro Obi, Masashi Sakuma, Norihiko Takeda, Yutaka Kano, Shigeru Toyoda, Toshiaki Nakajima

**Affiliations:** 1 Department of Cardiovascular Medicine, The University of Tokyo, Tokyo, JPN; 2 Department of Information Science and Technology, The University of Electro-Communications, Tokyo, JPN; 3 Department of Physical Therapy, Niigata University of Health and Welfare, Niigata, JPN; 4 Department of Cardiovascular Medicine, Dokkyo Medical University Hospital, Mibu, JPN; 5 Department of Medical KAATSU Training, Dokkyo Medical University Hospital, Mibu, JPN

**Keywords:** denervation, gene expression, microrna, muscle atrophy, vascular endothelial growth factor (vegf)

## Abstract

Background: Skeletal muscle atrophy is frequently caused by the disuse of muscles. It impacts quality of life, especially in aging populations and those with chronic diseases. Understanding the molecular mechanisms underlying muscle atrophy is crucial for developing effective therapies.

Objective: To investigate the roles of vascular endothelial growth factor (VEGF) and various microRNAs (miRNAs) in muscle atrophy using a mouse model of denervation (DEN)-induced disuse, and to elucidate their interactions and regulatory functions through comprehensive network analysis.

Methods: The right sciatic nerve of C57BL/6J mice (n=6) was excised to simulate DEN, with the left serving as a sham surgery control (Sham). Following a two-week period, wet muscle weight was measured. Total RNA was extracted from the tibialis anterior muscle for microarray analysis. Significant expression changes were analyzed via Kyoto Encyclopedia of Genes and Genomes (KEGG) pathway analysis and miRNet for miRNAs.

Results: Denervated limbs showed a significant reduction in muscle weight. Over 1,000 genes displayed increased expression, while 527 showed reductions to less than half of control levels. VEGF, along with specific miRNAs such as miR-106a-5p, miR-mir20a-5p, mir93-5p and mir17-5p, occupied central regulatory nodes within the gene network. Functional analysis revealed that these molecules are involved in key biological processes including regulation of cell migration, vasculature development, and regulation of endothelial cell proliferation. The increased miRNAs were subjected to further network analysis that revealed significant regulatory interactions with target mRNAs.

Conclusion: VEGF and miRNAs play crucial roles in the progression of skeletal muscle atrophy, offering potential targets for therapeutic interventions aimed at reducing atrophy and enhancing muscle regeneration.

## Introduction

Skeletal muscle atrophy, a condition characterized by a decrease in muscle mass and strength, is prevalent in multiple pathological conditions, including diabetes, obesity, and chronic diseases [1]. Prolonged inactivity and chronic inflammatory diseases are key factors in the development of skeletal muscle atrophy, which involves an imbalance in muscle protein synthesis and degradation that is often associated with oxidative and nitrosative stress [2] as well as inflammation and activation of proteasomal and autophagic systems [3,4]. The regulation of muscle protein balance during atrophy is influenced by disuse, aging, and disease, with a key role for satellite cells in muscle fiber atrophy [5,6]. Although this condition is complex, potential therapeutic strategies and intervention mechanisms for skeletal muscle atrophy are being explored [7]. Vascular endothelial growth factor (VEGF) is a key player in skeletal muscle angiogenesis and blood flow, and its expression levels increase after exercise [8]. Moreover, VEGF plays a crucial role in bone development and repair by directly influencing bone cell differentiation and function [9]. Local application of VEGF has been shown to improve muscle force, reduce scarring, and aid in muscle regeneration after acute trauma [10]. VEGF-expressing muscle stem cells enhance muscle repair through angiogenesis, regeneration, and fibrosis reduction [11], while impaired muscle regeneration is linked to delayed angiogenesis and VEGF production [12]. VEGF is also essential for adult muscle growth through impacts on inflammatory processes, satellite-endothelial cell interactions, and contractile protein accumulation [13] as well as exercise-induced capillary growth in human skeletal muscle [14].

microRNAs (miRNAs) are crucial for the regulation of muscle atrophy and thus are potential therapeutic targets [15-20]. Various biological processes involve miRNAs, including muscle differentiation and development [18,21]. Dysregulation of specific miRNAs that affect important signaling pathways is linked to the development of muscular atrophy [17]. Together, these findings suggest that miRNAs could be targeted to prevent or reverse muscular atrophy.

This study explored the roles of VEGF and miRNAs in skeletal muscle atrophy using an integrated network analysis approach. By analyzing gene expression profiles and identifying key regulatory molecules, we uncovered molecular interactions that could contribute to muscle atrophy and highlight potential targets for treatment.

## Materials and methods

Animal model and surgical procedure

We employed a denervation (DEN)-induced muscle atrophy model using male C57BL/6J mice (n=6). Under sterile conditions and anesthesia, the right sciatic nerves of eight-week-old mice were surgically excised to induce DEN, and the incision was sutured. The left sciatic nerve was exposed but not excised, serving as a sham surgery control (Sham). This bilateral approach allowed for direct comparisons within the same animal to minimize inter-animal variability. Post-surgery, mice were housed in a controlled environment at room temperature with a 12-hour light/dark cycle and ad libitum access to food and water. After a recovery period of two weeks that allowed for the onset and progression of muscle atrophy, the mice were euthanized, and the lower limbs were dissected for collection of skeletal muscles. All experiments were conducted under the guidelines established by the Physiological Society of Japan and were approved by the Niigata University of Health and Welfare Institutional Animal Care and Use Committee (#02120).

Muscle weight measurement

The wet weights of the harvested lower limb skeletal muscles were immediately recorded post-dissection. The weight measurements served as a primary indicator of muscle mass changes indicative of atrophy.

RNA extraction and gene expression analysis

Total RNA including small RNA was extracted from the tibialis anterior muscle using ISOGEN II (NIPPON GENE Co., Ltd., Tokyo, Japan) to recover high-quality RNA suitable for microarray analysis. We conducted a comprehensive gene expression profiling using microarray analysis that considered 24,878 mouse genes.

Normalization and expression analysis

To quantify relative changes in gene expression due to DEN, a comparative analysis was carried out. Gene expression levels in the DEN limb were compared to those in the sham-operated (Sham) limb, which was set as the control with a relative expression level of 1.

Statistical analysis of gene expression

Gene expression values were transformed after normalization to address technical variations. For each sample, the expression value was divided by the median expression value of all samples for each probe and converted to a Log2 scale. The statistical significance of differences in gene expression levels between groups (DEN vs. Sham) was calculated using Student’s t-test. Genes that showed a significant increase (≥ 2-fold) or decrease (≤ 0.5-fold) in expression were identified for further analysis.

Quantitative reverse transcription-polymerase chain reaction (RT-PCR)

Quantitative RT-PCR was performed to validate expression levels of critical genes, including VEGF mRNA, identified in the microarray analysis. This method provided a more sensitive and specific measurement of mRNA levels that helped to confirm the microarray data. For RT-PCR, complementary DNA (cDNA) was synthesized from a 40 ng/10-µl reaction of total RNA using a ReverTra Ace qPCR RT Master Mix (TOYOBO Co., Ltd., Osaka, Japan). Quantitative RT-PCR was performed using a KOD SYBR qPCR Mix (TOYOBO) and an Applied Biosystems 7300 Real-Time PCR System (Thermo Fisher Scientific, Waltham, MA, USA) as previously reported [22]. The reaction mixture was then subjected to PCR amplification with specific forward and reverse oligonucleotide primers for 40 cycles consisting of heat denaturation, annealing, and extension. PCR products were size-fractionated on 2% agarose gels and observed under blue LED light. The RNA level was analyzed relative to an internal control (GAPDH) used to normalize values for mRNA transcript quantity. The forward and reverse primer sequences for vascular endothelial growth factor A (VEGFA) were 5′-GACCCTGGCTTTACTGCTGTA-3′ and 5′-GTGAGGTTTGATCCGCATGAT-3′. The forward and reverse primer sequences for GAPDH were 5′-TGAAGGGTGGAGCCAAAAGG-3′ and 5′-GGAAGAGTGGGAGTTGCTGTTG-3′.

Pathway analysis

Genes that exhibited significant changes in expression between DEN and Sham limbs were identified based on stringent fold-change criteria; those with expression levels that increased by more than two-fold or were decreased by less than half were considered significant. These genes were included in a Kyoto Encyclopedia of Genes and Genomes (KEGG) pathway analysis using DAVID (https://david.ncifcrf.gov/) bioinformatics resources to examine biological pathways and networks affected by DEN. KEGG with DAVID bioinformatic analyses integrate information concerning gene networks, including metabolic and signaling pathways to provide insights into the functional implications of the observed gene expression changes. The analyses here focused on intermolecular interactions such as metabolism, signal transduction, and genetic information processing.

The STRING protein-protein interaction (PPI) and functional network

A PPI and functional network was constructed using the Search Tool for Retrieval of Interacting Genes (STRING) database (http://www.string-db.org/).

Microarray analysis of miRNA

Initial data analysis was done using results from a GeneChip™ miRNA 4.0 Array (Thermo Fisher Scientific, Inc.). Slides were washed and scanned on a GeneChip™ Scanner 3000 7G.

Evaluation of miRNA-hub gene interaction networks

Comprehensive miRNA analysis was performed using the miRNet database 2.0 (https://www.miRNet.ca/) tool. Those miRNAs that showed significant changes in expression, particularly those with a ≥1.8 fold-change, were identified and included in the analysis of target interactions. This approach allowed the mapping of regulatory networks of miRNAs and their impact on gene expression in the context of muscle atrophy.

Statistical analysis

All data are presented as mean ± standard deviation (SD). The comparison of means between groups was carried out using the Mann-Whitney U-test or Student's t-test. After normality examinations (Kolmogorov-Smirnov test or Shapiro-Wilk test), comparisons of means between groups were analyzed by a two-sided, unpaired Student’s t-test for normally distributed parameters or by the Mann-Whitney-U-test for non-normally distributed parameters. Associations among parameters were evaluated using Pearson correlation coefficients. All analyses were performed using Statistical Package for the Social Sciences (IBM SPSS Statistics for Windows, IBM Corp., Version 24.0, Armonk, NY). A p-value of 0.05 was regarded as significant.

## Results

Muscle weight measurement

**Figure 1:**
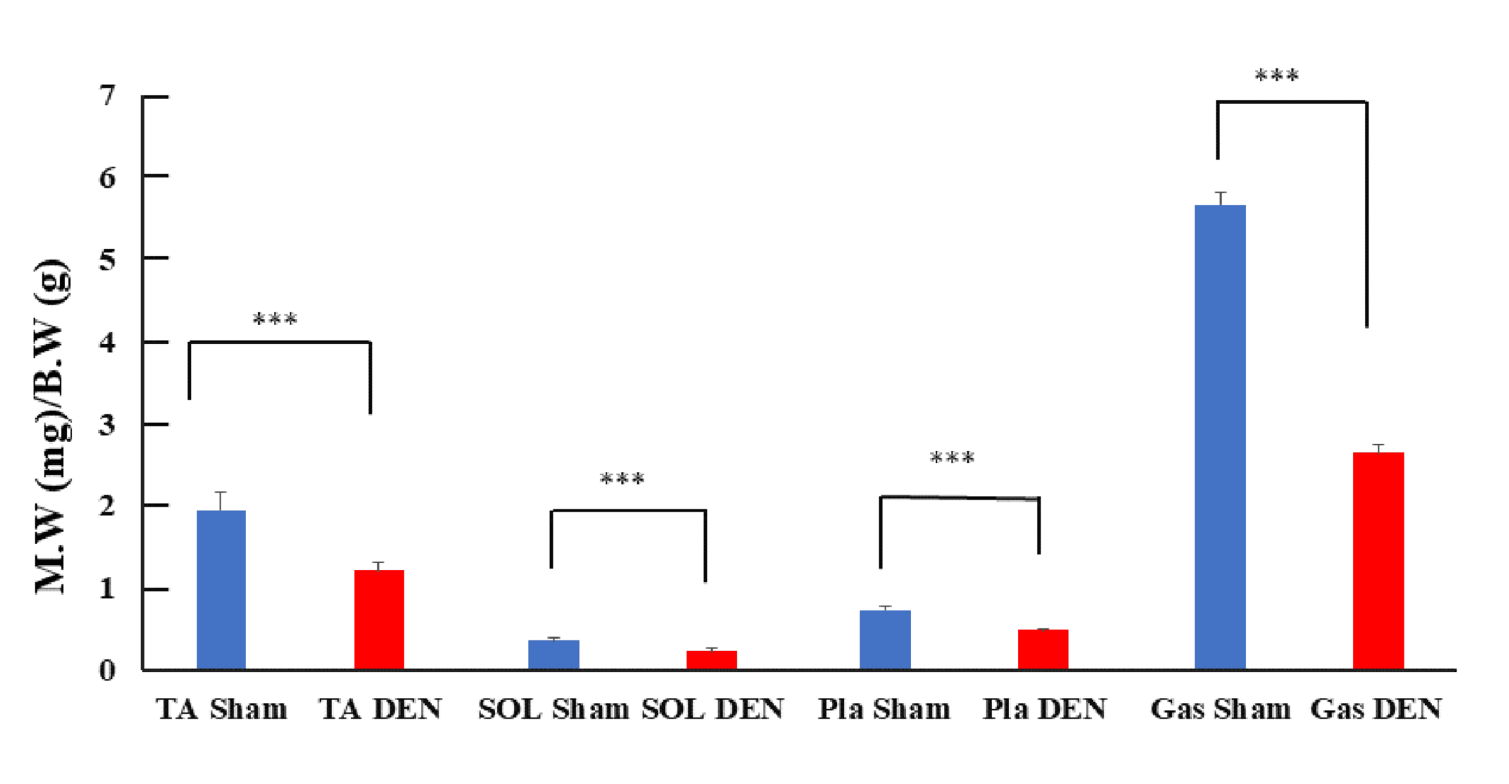
Comparison of wet muscle weights between DEN and Sham control limbs. Ratios of wet muscle weight (M.W) (mg) to body weight (B.W.) (g) are shown. Data are shown as mean ± standard deviation. *** p < 0.001; TA: tibialis anterior; SOL: soleus; Pla: plantaris; Gas: gastrocnemius; DEN: denervation

To assess the extent of muscle atrophy, the wet muscle weight of skeletal muscles from DEN and Sham-operated limbs was measured (Figure 1). The DEN limbs had a significant reduction in muscle mass compared to the Sham control, confirming that the DEN procedure successfully induced muscle atrophy. This measurement provides a direct and quantifiable indicator of the physical impact of nerve injury on muscle tissue. The muscle groups analyzed include the tibialis anterior (TA), soleus (SOL), plantaris (Pla), and gastrocnemius (Gas), categorized by fiber type: fast fiber for TA, Pla, and Gas, and slow fiber for SOL. Plotting the comparisons of Sham and DEN conditions for each muscle indicated a significant reduction in muscle weight across all tested groups following DEN (p-values < 0.001, asterisks). These findings demonstrate the profound impact of nerve injury on muscle mass and emphasize the vulnerability of both fast and slow fiber-containing muscles to atrophic changes under unloaded conditions.

Gene expression analysis

Gene expression microarray analysis revealed that 1033 genes had significantly upregulated expression (increased by more than two-fold) and 527 genes had significantly downregulated expression (less than half) in the DEN muscle compared to the Sham control. This significant shift in gene expression indicates profound molecular responses to DEN. The gene expression levels in the DEN limbs were quantified relative to the Sham control limbs, which were set to a relative expression level of 1.

Differential gene expression analysis

Gene expression analysis of DEN versus Sham control limb muscles revealed significant alterations across a broad array of genes that are pivotal for muscle function and repair. This comprehensive analysis highlights the stark contrasts in gene expression induced by muscle DEN, shedding light on the molecular disruptions that contribute to muscle atrophy.

Key findings for growth factors and vascular elements

**Figure 2:**
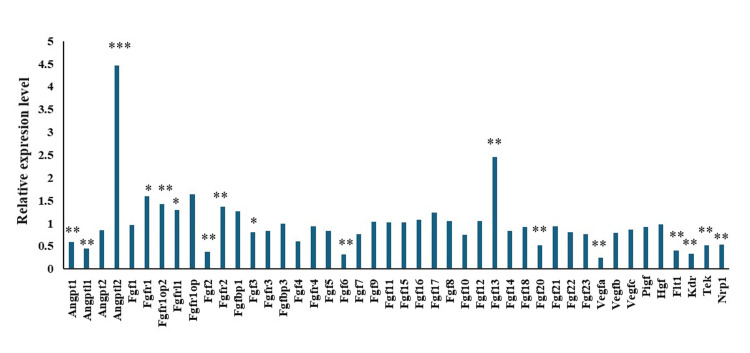
Comparison of mRNA levels for several critical growth factors and vascular elements in denervated versus control limb muscles. The relative gene expression levels in the denervated (DEN) limb are shown, compared to that in the sham-operated (Sham) limb, which was set as the control with a relative expression level of 1. *p < 0.05, **p < 0.01, ***p < 0.001.

The levels of mRNA for several critical growth factors and vascular elements showed notable changes. Expression of VEGF and its receptors, fms-like tyrosine kinase (Flt-1), and kinase insert domain-containing receptor/fetal liver kinase-1 (KDR/Flk-1), was significantly downregulated. VEGF, a major mediator of angiogenesis, exhibited a substantial decrease in expression (Figure 2, VEGFA: 0.25-fold), emphasizing its reduced capacity to support blood vessel growth under DEN conditions. Among the fibroblast growth factors (FGFs), FGF2 and FGF6 showed a marked reduction in expression levels (FGF2: 0.37-fold, FGF6: 0.31-fold), which could contribute to the diminished support for muscle tissue maintenance and repair. Meanwhile, the notable increase in FGF13 expression (2.45-fold) suggests that this factor could play a compensatory regulatory role in response to muscle injury. The mRNA levels of angiopoietins, which are crucial for vascular stability and remodeling, were also decreased, with angiopoietin-1 (0.58-fold) reflecting a broader downregulation of angiogenic signals. The downregulation of key factors involved in angiogenesis and cell proliferation likely contributes to the impaired regeneration observed in denervated muscle tissue. This decrease in essential growth factors and receptors not only compromises muscle cell survival and repair but also impacts the overall vascular integrity that is important for sustaining muscle health.

Quantitative RT-PCR and microarray analysis of VEGF mRNA expression

**Figure 3:**
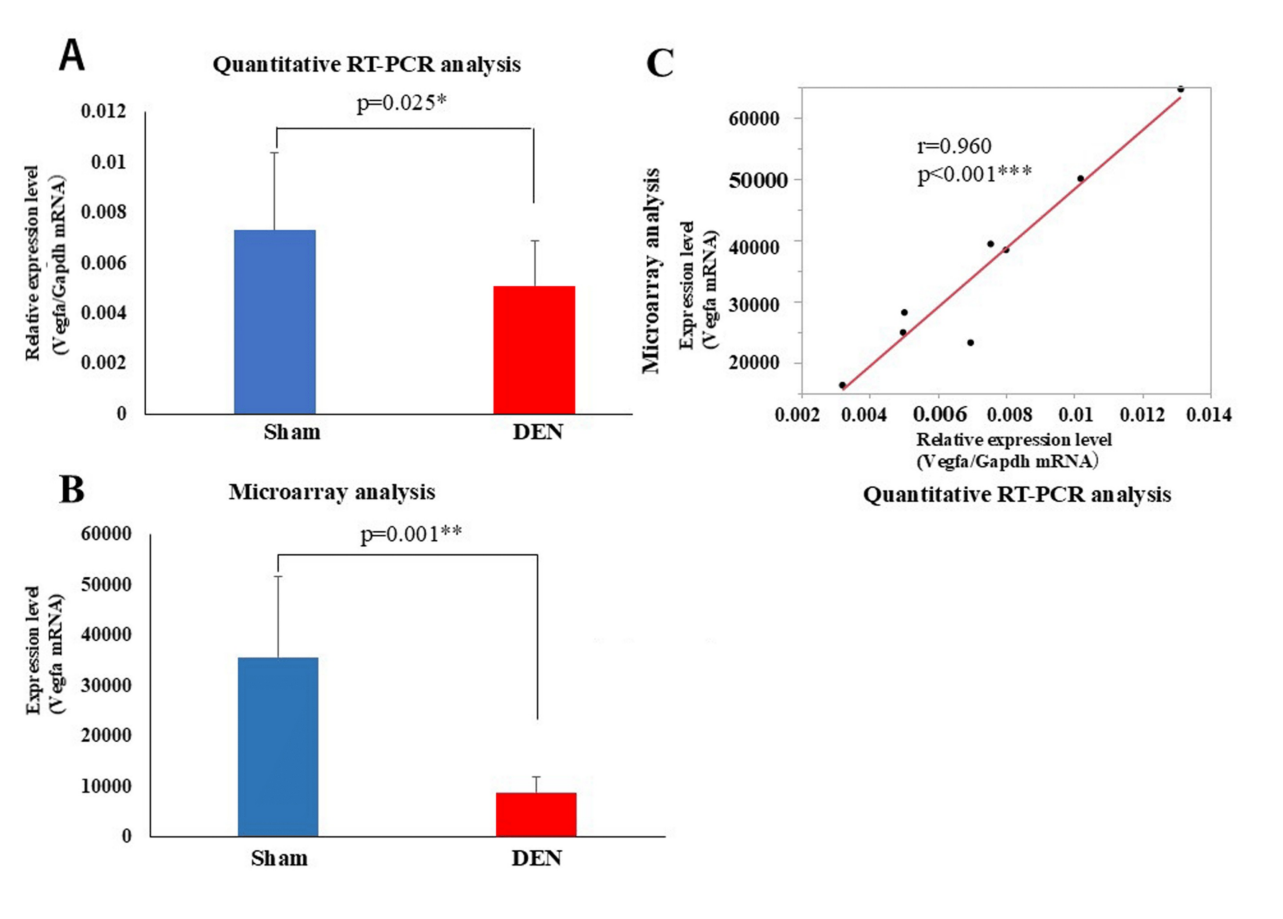
Comparison between quantitative RT-PCR and microarray analyses to assess VEGFA mRNA expression levels. (A) Quantitative RT-PCR and (B) microarray data. Data are shown as mean ± standard deviation. *p <0.05, **p < 0.01 (C) Correlation between RT-PCR and microarray data. r-value and p-value are shown ***p < 0.001. RT-PCR: reverse transcription-polymerase chain reaction; VEGFA: vascular endothelial growth factor A; DEN: denervation

Quantitative RT-PCR and microarray analyses were performed to assess the expression levels of VEGFA mRNA in TA muscles from DEN and control (Sham) conditions (Figure 3). Quantitative RT-PCR (Figure 3a) results showed a significant reduction in VEGFA mRNA levels in the denervated muscles compared to controls, with quantitatively lower expression levels in the DEN group (p = 0.001**). The microarray data (Figure 3b) corroborated these findings, showing a similar pattern of downregulation in VEGF expression in denervated muscles. The notably high correlation between the RT-PCR and microarray data (Figure 3c, r = 0.960, p < 0.001) reinforces the reliability of the observed changes in gene expression. Both the RT-PCR and microarray data clearly demonstrate the significant reduction in VEGF mRNA in denervated muscles.

KEGG pathway analysis of genes with decreased expression in denervated limbs

**Figure 4:**
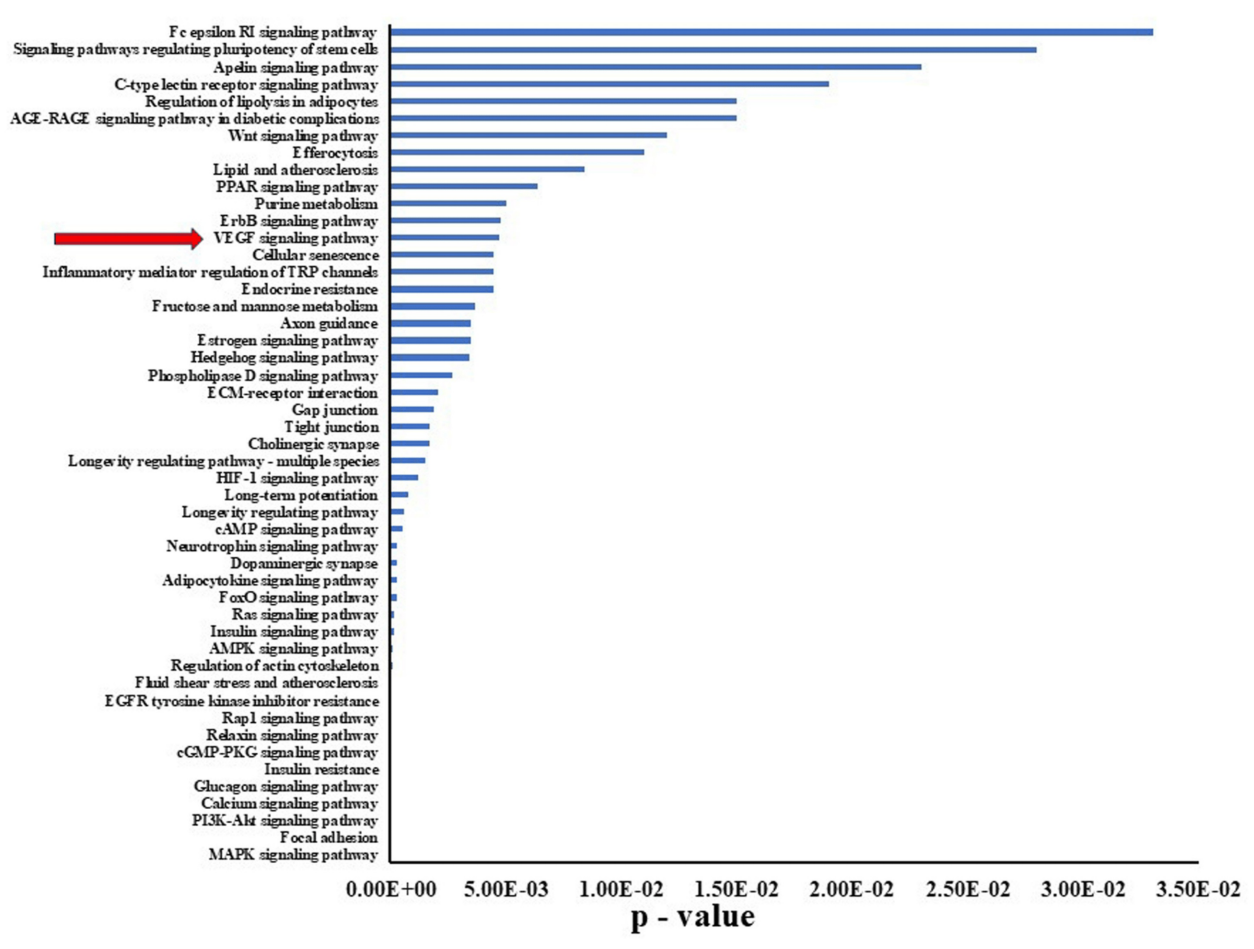
KEGG pathway analysis of genes with decreased expression in denervated limbs. P-values for genes that exhibited significant decreases in expression in denervated limbs (DEN) relative to Sham control limbs are shown. The VEGF signaling pathway is highlighted by a red arrow. KEGG: Kyoto Encyclopedia of Genes and Genomes; VEGF: vascular endothelial growth factor

**Figure 5:**
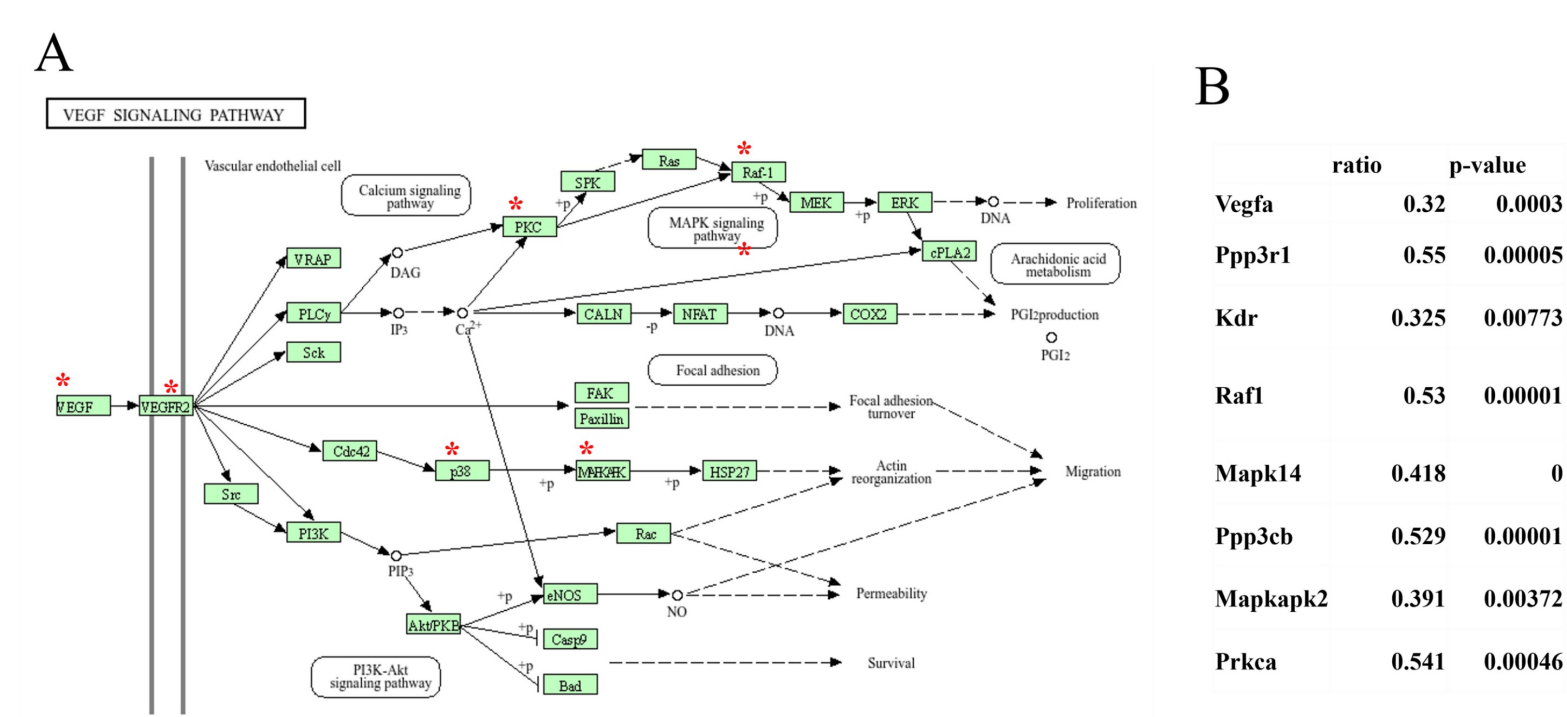
Effects of denervation-induced muscle atrophy on expression of genes in the VEGF signaling pathway. (A) KEGG pathway analysis of the VEGF signaling pathway in denervation-induced muscle atrophy. Asterisks (*) indicate genes having significantly decreased expression in denervated limbs (DEN) relative to that of sham-operated limbs, which was set to a relative expression level of 1. (B) Relative expression level of genes with decreased expression. KEGG: Kyoto Encyclopedia of Genes and Genomes; VEGF: vascular endothelial growth factor

KEGG pathway analysis of the 527 genes that exhibited a decrease in expression by more than half relative to control samples was carried out to delineate the biological pathways that were significantly impacted by the observed downregulation. Various pathways were affected by this decrease in gene expression, encompassing areas related to cellular processes, metabolic pathways, and critical signaling pathways, including the VEGF signaling pathways (Figure 4). A pathway diagram highlights key components that experienced notable downregulation (Figure 5A). The key signaling molecules such as VEGF receptor 2 (VEGFR2), phospholipase C gamma (PLCγ), and growth factor receptor-bound protein 2 (Grb2) are marked with red asterisks, indicating their critical roles in vascular permeability, cellular proliferation, and survival. Downregulation of these components could potentially disrupt angiogenesis and cellular repair mechanisms that are crucial for maintaining tissue integrity under stress conditions. A quantitative summary of changes in gene expression within the VEGF signaling pathway was also made that includes notable genes such as VEGFA, Ppp3r1, and Raf1, which all showed significant decreases in expression (ratios of 0.32, 0.55, and 0.53, respectively; p-values <0.05; Figure 5B). These results underscore the extent of disruption caused by the decreased expression of these genes and directly link the molecular alterations with potential functional consequences in the signaling pathway.

Microarray analysis of miRNA expression

**Figure 6:**
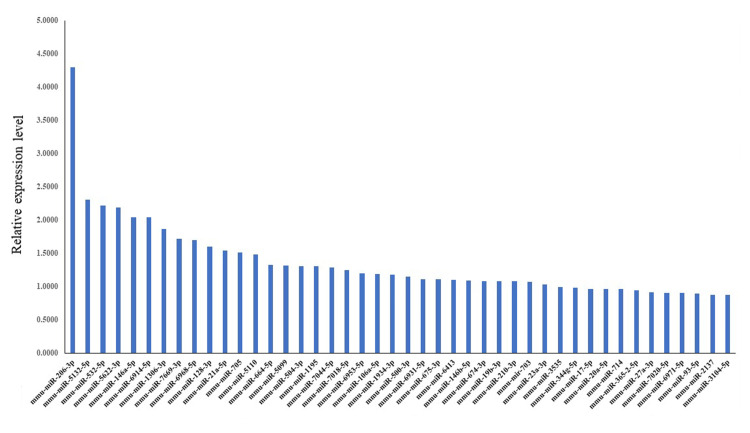
miRNAs having significant change in expression following denervation. Blue bars represent relative miRNA expression levels. miRNA: microRNA

We next carried out a microarray analysis to identify miRNAs that exhibited differential expression in response to DEN. A total of 44 miRNAs were found to have significant differences in expression levels between denervated and control limbs. These miRNAs showed a Log2 fold change greater than 1.8, and each maintained a p-value of less than 0.05, indicating statistically significant upregulation (Figure 6).

Centrality analysis and network construction

**Figure 7:**
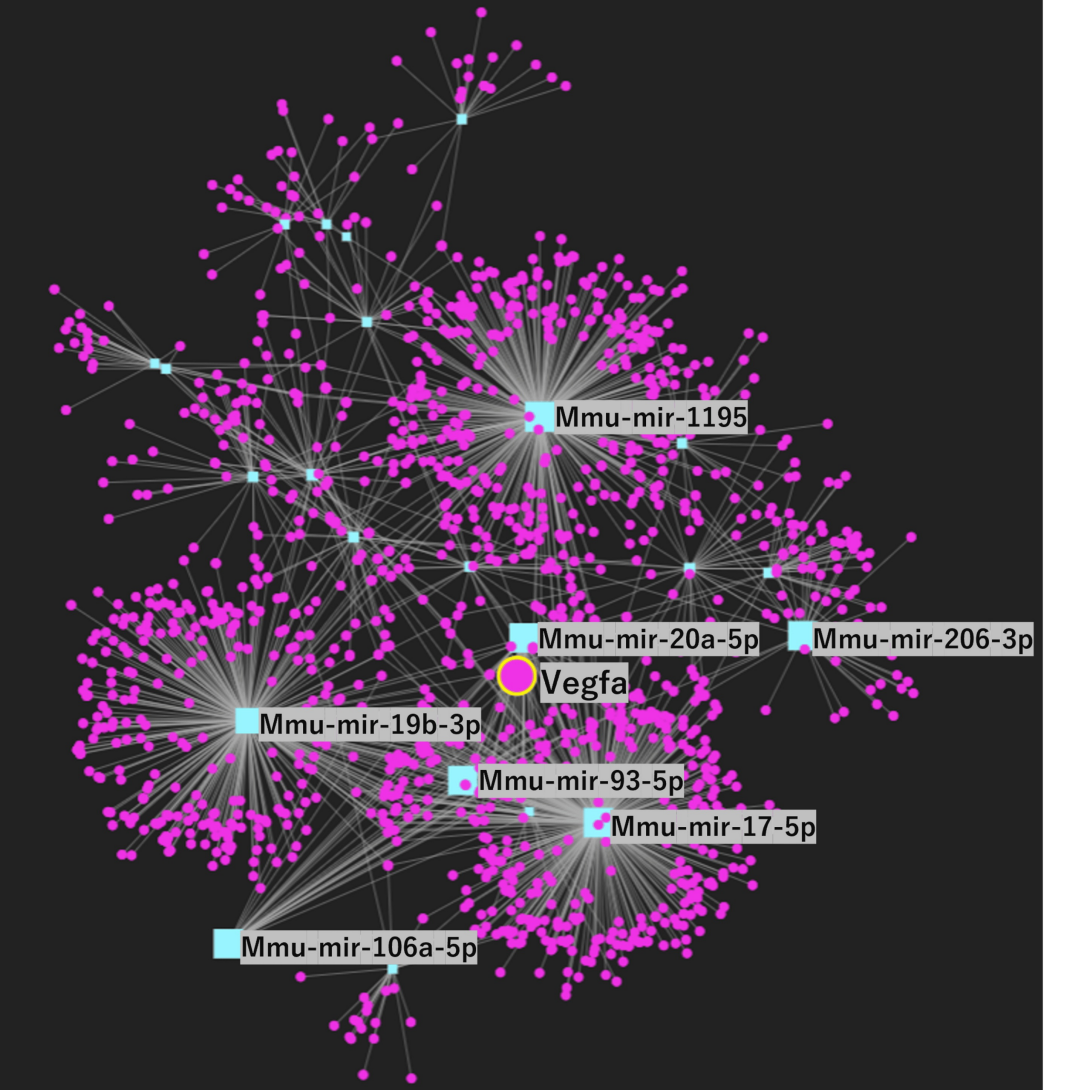
Network analysis of miRNAs related to VEGFA. Purple circles represent gene. VEGFA gene is shown by large teal circle. Blue squares represent miRNA. The key miRNAs are illustrated by large teal squares. Gray connecting lines present interactions. miRNA: microRNA; VEGF: vascular endothelial growth factor

A network analysis of the 44 miRNAs that displayed a significant increase in expression in denervated levels was carried out using miRNet. Among these miRNAs, four miRNAs were closely related to VEGF regulation and are involved in intricate relationships within a broader regulatory network that influences VEGF pathways (Figure 7). Centrality measures were used to construct a comprehensive gene network involving VEGF and specific miRNAs including miR-106a-5p, miR-mir20a-5p, mir93-5p, and mir17-5p as key hub nodes. These nodes have a high degree of centrality, betweenness centrality, and closeness centrality, indicating their pivotal roles in the regulatory network. The identification of these key nodes underscores the importance of VEGF and miRNAs in the molecular mechanisms that drive muscle atrophy. Further network analysis using miRNet with the four miRNAs that were directly associated with VEGFA revealed a significant correlation among three specific miRNAs miR-106a-5p, miR-mir20a-5p, and mir17-5p, which formed a new distinct network.

KEGG pathway analysis of targeted miRNAs

**Figure 8:**
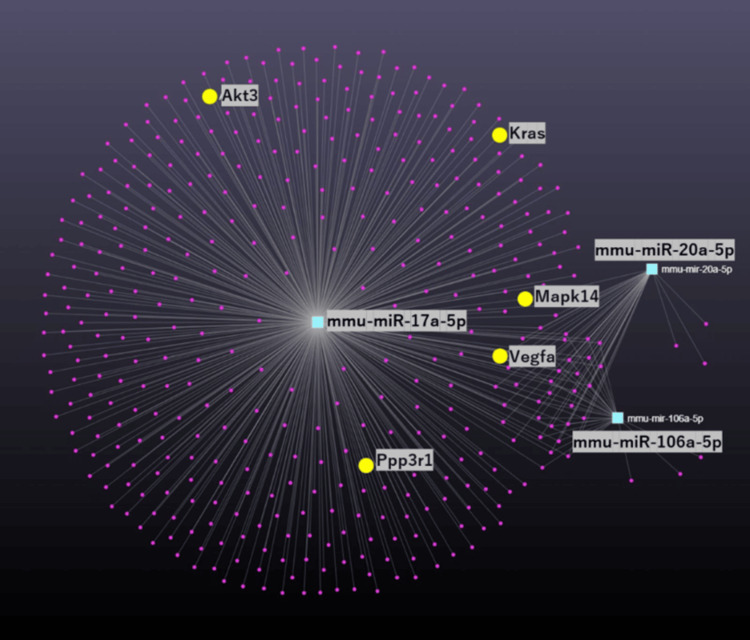
Pathway analysis of targeted miRNAs. Purple circles represent genes. Teal squares represent miRNA. Yellow circles present key genes. Gray connecting lines present interactions. miRNA: microRNA

Further KEGG pathway analysis of the three VEGF-related miRNA networks was conducted to delineate the roles of these miRNAs in cellular signaling pathways. These three miRNAs were confirmed to be involved in the VEGF signaling pathway and to affect five key genes: Akt3, Kras, Mapk14, Ppp3r1, and VEGFA, which are integral to this pathway (p = 0.01; Figure 8). These genes all are critical for the control of angiogenesis and muscle repair processes.

Gene ontology (GO) biological process analysis

GO analysis of differentially expressed genes revealed significant enrichment in processes associated with tissue integrity and repair as well as striated muscle tissue development, cell development, cell migration, vasculature development, and endothelial cell proliferation (Table 1). The affected genes are known to be centrally involved in enhancing tissue regeneration and vascular health and would be important for mounting an effective response to muscle atrophy and for promoting recovery.

**Table 1:** GO biological process analysis GO: Gene ontology

Process	Hits	p-value
Striated muscle tissue development	29	5.83e-7
Cell development	74	0.0000043
Regulation of developmental process	72	0.000289
Positive regulation of cell migration	17	0.000361
Vasculature development	31	0.000511
Regulation of cell differentiation	51	0.00174
Regulation of endothelial cell proliferation	36	0.00336

STRING PPI and functional network of the VEGFA gene

**Figure 9:**
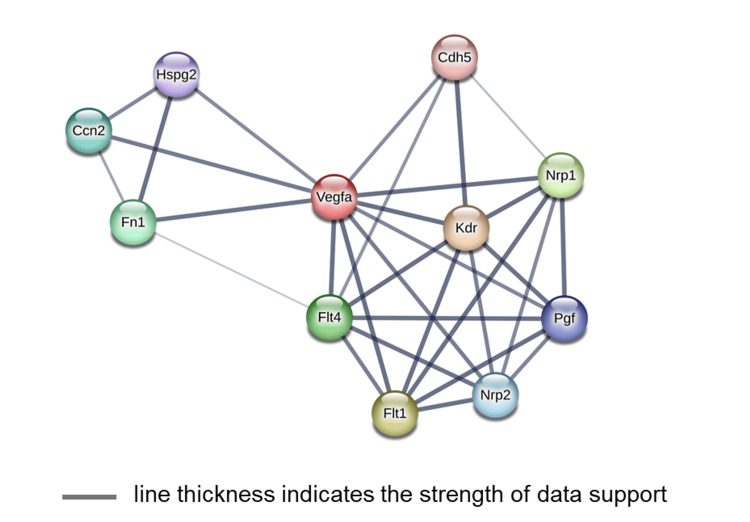
STRING protein-protein interactions (PPI) and functional network of the VEGFA gene STRING: Search Tool for Retrieval of Interacting Genes; VEGFA: vascular endothelial growth factor A

We used the STRING database to elucidate the protein interaction network associated with VEGFA. This analysis highlighted a complex network of proteins involved in the signaling pathways that can potentially influence VEGF (Figure 9).

Integrating STRING database findings with miRNet analysis

**Figure 10:**
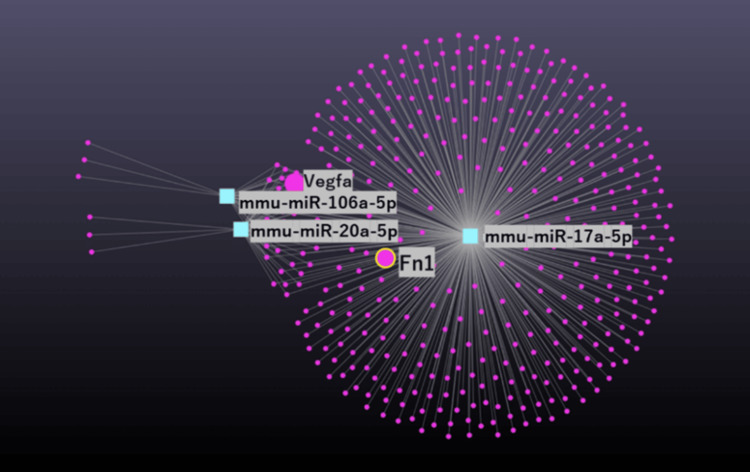
miRNet network analysis of miRNAs related to Fn1. Purple circles represent genes. Teal squares represent miRNA. Gray connecting lines present interactions. miRNA: microRNA

A subsequent analysis using miRNet focused on proteins identified from the STRING database results to ascertain their presence within miRNA regulatory networks. Notably, Fibronectin 1 (Fn1), a protein highlighted in the STRING database, was also found to be a component of the network derived using miRNet (Figure 10). This discovery highlights potential regulatory interactions involving Fn1 and its influence over muscle atrophy pathways mediated by VEGFA.

Significant decrease in Fn1 mRNA expression in denervated limbs

Microarray analysis confirmed that Fn1 mRNA levels in DEN limbs were significantly decreased relative to Sham control limbs (mean ratio 0.592, p = 0.03). The decrease in Fn1 mRNA substantiates its involvement in the cellular mechanisms that may contribute to muscle degradation processes associated with VEGF signaling. These findings collectively suggest that Fn1 is a crucial gene within the VEGF signaling network affecting muscle atrophy. Collectively, the evidence from protein network analysis (STRING), regulatory network examination (miRNet), and mRNA expression patterns (microarray) point to a significant role for Fn1 in the pathophysiology of muscle degeneration. Moreover, the pivotal role of Fn1 suggests its potential as a target for therapeutic interventions to mitigate muscle atrophy linked to impaired VEGF signaling.

## Discussion

This study provides evidence that VEGF and specific miRNAs play critical roles in skeletal muscle atrophy. The downregulation of key angiogenesis-related genes and their receptors in denervated muscle suggests a significant impairment in angiogenic processes that are crucial for muscle regeneration and repair. The marked decrease in VEGF mRNA expression after DEN as detected by both quantitative RT-PCR and microarray analyses points to a significant disruption in the molecular pathways that support muscle angiogenesis and recovery. VEGF is crucial for recruiting blood vessels to the injury sites, and its reduced expression could hinder the delivery of oxygen and nutrients needed for effective muscle repair and regeneration. These findings underscore the molecular impact of DEN on muscle tissues, specifically through the downregulation of essential growth factors like VEGF. This reduction likely contributes to the atrophy and impaired healing observed in denervated muscle, making VEGF a critical target for therapeutic strategies aimed at enhancing muscle recovery after nerve injuries. The identification of VEGF as a central hub in the gene network underscores its importance in maintaining muscle integrity and function. The mammalian VEGF family consists of five different polypeptides: VEGFA, VEGFB, VEGFC, VEGFD, and placental growth factor (PlGF). VEGFA, which was originally identified as a vascular permeability factor (VPF), was the first member of this family to be characterized [22]. VEGFA is the fundamental regulator of vascular growth and the key target of therapeutic angiogenesis approaches. It is the most important family member, controlling blood vessel growth in both physiological and pathological angiogenesis. VEGFB and PlGF play more accessory or tissue-specific roles, while VEGFC and VEGFD mostly regulate lymphatic vessels [8]. In this study, the expression levels of VEGFB, VEGFC, and PIGF did not significantly change.

A body of research highlighted the crucial role of miRNA responses to muscle atrophy. These small non-coding RNA molecules have been shown to modulate gene expression, particularly in the context of muscle maintenance and degradation [15]. Dysregulation of miRNAs has been linked to the development of muscular atrophy, affecting important signaling pathways [17]. These findings suggest that miRNAs may serve as potential therapeutic targets in muscle atrophy.

A previous meta-analysis [23] identified key miRNA-mRNA interactions in denervated muscle atrophy, including miR-1/VEGFA and miR-206/VEGFA. Other studies also explored these interactions [15,24] and highlighted the role of miRNAs in regulating muscle atrophy with specific involvement of miRNA-206 and miRNA-21 in the atrophy pathways. The importance of VEGF in maintaining muscle integrity and function was also underscored by Moimas et al. [25], who found that VEGF gene therapy attenuated DEN-related muscle atrophy. This finding is consistent with that of Wagner [8] and Ochoa et al. [12], who demonstrated the critical role of VEGF in skeletal muscle angiogenesis and blood flow, and its restoration in muscle regeneration. These studies collectively provide compelling evidence for the critical roles of VEGF and specific miRNAs in skeletal muscle atrophy.

Our miRNA analysis revealed significant interactions between miRNAs and their target mRNAs, highlighting regulatory roles for miRNAs in muscle atrophy. miR-106a-5p, miR-mir20a-5p, mir93-5p, and mir17-5p in particular were identified as key regulators, likely due to their ability to modulate the expression of multiple target genes involved in muscle maintenance and degradation. Further analysis of miRNAs that had ≥1.8-fold changes in expression provided additional insights into their critical roles in gene regulation during muscle atrophy. The miR-17 family, including miR-20a-5p, miR-106a-5p, and miR-20b-5p, plays a crucial role in muscle development and function. These miRNAs are involved in myoblast proliferation and differentiation, with miR-20a-5p and miR-20b-5p promoting differentiation and repressing proliferation [26]. On the other hand, miR-106a-5p inhibits myogenesis by targeting PIK3R1 and modulating the PI3K/AKT signaling pathway [27]. The muscle-specific miRNAs miR-1, miR-133, and miR-206 are also critical for muscle development and function, with miR-1 and miR-206 regulating satellite cell proliferation and differentiation [28,29]. Meanwhile, miR-206 is highly expressed in newly formed muscle fibers, indicating its potential role in muscle regeneration and maturation [30]. The expression of miR-1 and miR-133a is decreased during skeletal muscle hypertrophy [31], and these miRNAs are induced during the myoblast-myotube transition by myogenic factors such as myogenin and MyoD [32]. Further research is needed to explore the potential of miRNAs as therapeutic targets in muscle atrophy, particularly in the context of cachexia [33].

Functional enrichment analysis provides a deeper understanding of the molecular mechanisms underlying muscle atrophy, with a focus on the involvement of VEGF in vasculature development, cell migration, and cell proliferation, and the identification of specific miRNAs linked to regulatory pathways crucial for muscle health [15,27,34,35]. These findings suggest that targeting VEGF and these specific miRNAs could be a promising therapeutic approach for preventing or mitigating muscle atrophy.

As a key regulator of angiogenesis, VEGF binds to its receptors Flt-1 and KDR/Flk-1, which are selectively expressed in vascular endothelium [36]. The binding of VEGF to these receptors triggers endothelial cell growth and angiogenesis [37]. KDR/Flk-1 is particularly important in VEGF-induced endothelial cell proliferation and angiogenesis [38]. FGF2 and FGF6 also play crucial roles in angiogenesis [39]. The VEGF-VEGFR system is a crucial target for anti-angiogenic therapy in cancer [40]. Angiopoietin-1 and angiopoietin-2, which act through receptors Flt-1 and Tie-2, also play a role in tumor angiogenesis [41]. Fn1 and its receptor Fn14 also play crucial roles in muscle development and function. Fn1 is essential for proper muscle fiber organization and length regulation during embryonic development [42]. In addition, Fn1 mediates mechanical coupling between skeletal muscle contraction and local vasodilation, influencing blood flow regulation [43]. Several studies highlighted the intricate relationship between VEGF signaling and Fn in muscle and endothelial cell function. Fn, a major extracellular matrix protein, interacts with VEGF and its receptors to enhance VEGF-induced angiogenesis and cell survival [44]. These findings underscore the importance of VEGF-Fn interactions in vascular development and potential therapeutic targets for angiogenesis-related disorders [45] and suggest their potential as promising targets for therapeutic interventions to mitigate muscle atrophy and promote muscle regeneration.

This study has several limitations. In this study, we utilized a two-week timeframe post-DEN based on established models demonstrating that significant molecular changes indicative of muscle atrophy, including alterations in the VEGF pathway, become apparent within this period. However, the dynamics of VEGF and its related signaling pathways in response to muscle atrophy warrant further exploration. Future studies should consider sequential time points to map the progressive changes in VEGF expression and activity. However, this would provide a more comprehensive understanding of the temporal aspects of VEGF-mediated signaling during the early and advanced stages of muscle atrophy, helping to pinpoint critical windows for therapeutic intervention.

Further experimental validation of the interactions predicted using publicly available data is needed. Additional investigations involving in vivo studies are needed to verify the insights provided by network analyses in order to fully understand their contributions to muscle atrophy.

## Conclusions

This study demonstrates that VEGF and specific miRNAs such as miR-106a-5p and miR-20a-5p are critical in regulating skeletal muscle atrophy, identified through detailed network analysis in a mouse model. These molecules are pivotal in modulating key biological processes including regulation of cell migration, vasculature development, and regulation of endothelial cell proliferation, which are essential for muscle maintenance during atrophy. The research underscores the therapeutic potential of targeting these miRNAs and VEGF to mitigate muscle atrophy and enhance regeneration. Future efforts should focus on clinical validation of these targets to develop effective treatments for muscle atrophy.
